# A retrospective review of the Honduras AIN-C program guided by a community health worker performance logic model

**DOI:** 10.1186/s12960-016-0115-x

**Published:** 2016-05-06

**Authors:** Daniela C. Rodríguez, Lauren A. Peterson

**Affiliations:** Department of International Health, Johns Hopkins Bloomberg School of Public Health, 615 N. Wolfe Street, Rm. E-8612, Baltimore, MD 21205 USA; Abt Associates, Bethesda, MD USA

**Keywords:** Community health workers, Performance, Community-based program, Malnutrition, Honduras

## Abstract

**Background:**

Factors that influence performance of community health workers (CHWs) delivering health services are not well understood. A recent logic model proposed categories of support from both health sector and communities that influence CHW performance and program outcomes. This logic model has been used to review a growth monitoring program delivered by CHWs in Honduras, known as *Atención Integral a la Niñez en la Comunidad* (AIN-C).

**Methods:**

A retrospective review of AIN-C was conducted through a document desk review and supplemented with in-depth interviews. Documents were systematically coded using the categories from the logic model, and gaps were addressed through interviews. Authors reviewed coded data for each category to analyze program details and outcomes as well as identify potential issues and gaps in the logic model.

**Results:**

Categories from the logic model were inconsistently represented, with more information available for health sector than community. Context and input activities were not well documented. Information on health sector systems-level activities was available for governance but limited for other categories, while not much was found for community systems-level activities. Most available information focused on program-level activities with substantial data on technical support. Output, outcome, and impact data were drawn from various resources and suggest mixed results of AIN-C on indicators of interest.

**Conclusions:**

Assessing CHW performance through a desk review left gaps that could not be addressed about the relationship of activities and performance. There were critical characteristics of program design that made it contextually appropriate; however, it was difficult to identify clear links between AIN-C and malnutrition indicators. Regarding the logic model, several categories were too broad (e.g., technical support, context) and some aspects of AIN-C did not fit neatly in logic model categories (e.g., political commitment, equity, flexibility in implementation). The CHW performance logic model has potential as a tool for program planning and evaluation but would benefit from additional supporting tools and materials to facilitate and operationalize its use.

**Electronic supplementary material:**

The online version of this article (doi:10.1186/s12960-016-0115-x) contains supplementary material, which is available to authorized users.

## Background

Given the existing and increasing efforts to deliver health services through community health workers (CHWs), critical questions remain about their performance and how to improve it to reach the greatest health gains possible. Many studies on CHW performance have focused on aspects of service delivery or health outcomes [1–3], but not on the factors that specifically influence performance. A recent evidence summit on CHW performance hosted by the US government concluded that while it was plausible that support, both from health systems and communities, would positively influence CHW performance, the relationship between these is not well understood because research on potential support activities (individually or in combination) have not been frequently or adequately investigated [4].

Community and health system support activities with potential to influence performance identified at the Summit, such as local health committees, community participation, drug availability, and support from government entities, reflect those under study elsewhere [5–7]. In fact, two studies are specifically focusing on performance-related interventions. Kallander et al. have developed a protocol to test two intervention packages to improve CHW performance and retention in Uganda and Mozambique: (i) an mHealth package with communication, motivational messages, and phone-based data and supervision activities, and (ii) a community engagement package with village health clubs conducting activities to improve CHW status, standing, and demand for services [7]. Vareilles and colleagues are conducting a realist evaluation to identify factors influencing performance of community health volunteers in an immunization program in Uganda [8].

**Fig. 1:**
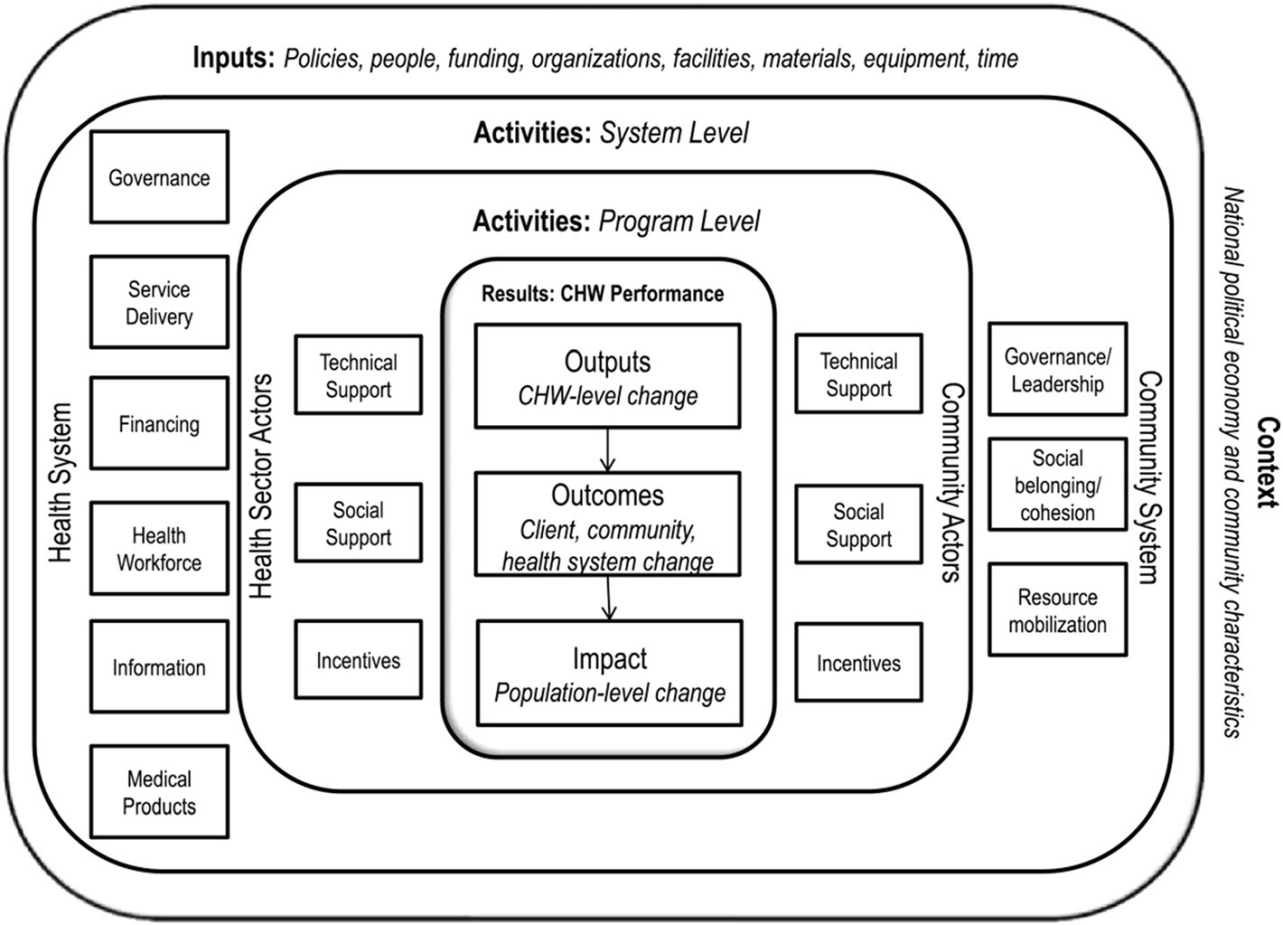
CHW performance logic model. Source [9], reprinted with permission of authors

Building on the work from the Summit [4], Naimoli and colleagues developed a generic logic model for CHW performance that incorporates multiple dimensions (Fig. 1) [9]. At the center of the model, three measures of CHW performance results are highlighted: outputs of CHW-level change; client, community, and health systems outcomes attributable to CHWs; and population-level health impacts attributable to CHWs. These performance measures are surrounded and driven by program-level activities by actors in the health sector and the community, which are in turn affected by systems-level activities from both health and community systems. The overall processes are underpinned by inputs and contextual factors.

The objective of this study was to conduct a retrospective review of a CHW program using the generic logic model for CHW performance and identify the factors contributing to the program’s success in improving health outcomes. The program under review is a national community-based health and nutrition program focused on growth monitoring in Honduras known as *Atención Integral a la Niñez en la Comunidad* or Integrated Child Health Program in the Community (AIN-C), which is described in detail below.

### AIN-C program in Honduras

**Fig. 2:**
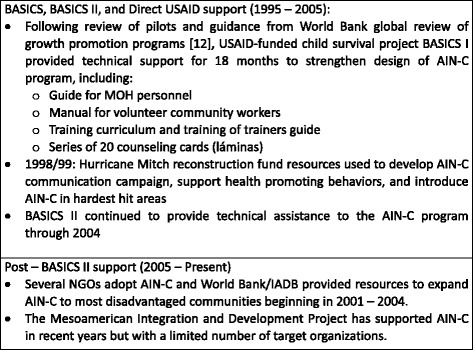
AIN-C program history. Source [10, 14]; INT 07.30.14; INT 08.13.14; INT 08.15.14

In the late 1980s/early 1990s, the Ministry of Health (MOH) in Honduras suspected that persistent malnutrition was a key factor in static mortality rates and developed the Integrated Care of the Child (AIN) program to detect growth faltering in health facilities. A review conducted in 1994 suggested that services should be taken beyond the facility level [10, 11]. A community-based approach (AIN-C) was piloted and determined to be the best way to reach rural families. From 1995 through 2005, USAID’s BASICS program supported the development and expansion of the AIN-C program. Figure 2 outlines the evolution of support provided to AIN-C from the BASICS program as well as the post-BASICS period.

AIN-C was targeted at children under five and was supported by volunteer CHWs known as *monitoras* who conducted community-based growth monitoring program (GMP) to detect faltering early and promote feeding strategies. *Monitoras* were selected by the community. The program had an unusual design in that it employed teams of *monitoras* to share responsibilities in each community at any given time [10]. The number of *monitoras* on a team varied between two and five, but three was the average [12], and teams were required to have at least one female member and one literate member in order for the team to perform effectively (INT 07.30.14). These arrangements were intended to manage CHW turnover.

The *monitoras* provided monthly monitoring for children under two and followed up with households that missed monthly monitoring appointments [12, 13]. They also provided counseling on child nutrition, care of common illnesses, and referrals to health center nurses for children under five with counseling cards developed after extensive formative research [10, 12, 13]. The program was designed to be flexible in implementation and *monitoras* were allowed to hold growth monitoring sessions in the format most suitable to their community (e.g., one session monthly, four weekly sessions per month). The program was also designed to encourage active community participation and evidence-based decisions with *monitora* teams engaging communities in discussion about key issues that had implications for the nutrition and health status of local children [13].

Monitoring sessions involved weighing each child, tracking their growth on a simple ledger, and counseling caregivers as needed. The data collected were to be used (i) by the *monitoras* to trigger dialogue with caregivers and inform individualized counseling with counseling cards, (ii) by the community to measure progress and identify impediments to growth, and (iii) by health system actors to measure outcomes and improve the program [11].

*Monitoras* were supervised directly by the health center nurse auxiliary during growth monitoring sessions, and as *monitoras* built knowledge and skills, they were given increased independence and responsibility. Health sector nurse supervisors also visited but more infrequently. Further, monthly meetings were held at the health center with other community volunteers to review progress, receive training, and restock medicines and supplies [14].

AIN-C was designed to roll out to 60 communities per year, with all communities covered nationwide within 6 years [12]. Six disadvantaged departments were targeted at first: Comayagua, Copán, Intibucá, La Paz, Lempira, and Ocotepeque.

A midterm evaluation by BASICS in 2000 suggested that AIN-C communities were more likely to know about the program, participate in growth monitoring, and attend weighing sessions consistently than control communities [15]. High rates of participation and attendance continued through the final evaluation; however, awareness that inadequate weight gain was a sign of poor growth was not significantly higher [11]. A cost analysis estimated that AIN-C had a long-term annual, recurrent cost per child under five of $2.73, and the average direct cost per child of an AIN-C session was approximately 11 % of the direct cost of a single MOH facility-based consultation [12, 14].

## Methods

This study was conducted as a retrospective documentary review, which was complemented by in-depth interviews with knowledgeable respondents. A total of 30 documents were obtained, including World Bank and USAID reports, MOH documents and policies, national health surveys, and presentations (see Additional file 1). Documents were obtained through USAID contacts, web searches, and documents shared directly by interviewees.

Documents were coded systematically using a codebook based on the CHW performance logic model. A code for each logic model component was defined and applied across documents. An additional code of “Other” was used to code any sections of text that did not fit the predetermined categories. At the end of coding, study authors reviewed these portions of text together to determine whether an existing code could be used. The documents were coded using qualitative software NVivo (QSR International).

Four in-depth interviews were conducted with individuals intimately familiar with the development and implementation of the AIN-C program, including former MOH officials, USAID personnel, and contractors. The first interviewee was identified by contacts at USAID, and she then suggested additional interviewees to contact. One interview was conducted face-to-face and three were telephone-based. Interviews lasted between 30 and 60 min. The telephone interviews were recorded, but not transcribed, and extensive notes were taken for all interviews. The interviews were unstructured and were used to fill gaps in knowledge from the document review and confirm preliminary analyses. Of the total 30 documents reviewed, seven were provided by interviewees.

Analysis was iterative. Coded output was reviewed by code and across logic model categories to identify factors contributing to the success of the AIN-C program as well as critical areas that presented challenges. Gaps in timeline or understanding as well as issues of clarity were explored with respondents, and analyses were subsequently refined.

No ethics review was sought for this project as it was a desk review from existing documentation, with interviewees reflecting on prior work experiences.

## Results

Most of the available evidence focused on the period during which BASICS supported the AIN-C program, with minimal publicly available information about the current status of AIN-C. Current policy documents from the MOH [16–18] mention AIN-C as a program requiring continued commitment but there were few specifics about the current program, how it is being implemented or how it is being financially supported. Thus, the results below focus on the BASICS-supported period of AIN-C (1995–2005) organized by the categories of the CHW performance logic model.

### Context

Prior to AIN-C, there had been limited improvements in human development in Honduras. Gains had been made in education and health with little progress in malnutrition [16]. In an effort to improve equity, it was decided that AIN-C would target communities with the greatest need: (i) those with higher prevalence of acute respiratory infection (ARI) and diarrhea in children under five, (ii) those where chronic malnutrition was a persistent problem, and (iii) rural, disadvantaged, primarily indigenous populations [10, 15].

Other broader contextual factors fed into the development of the program. First, Honduras had a history of volunteerism, especially for health, which provided a backbone for establishing a volunteer cadre of health workers [12]. Second, despite taking place after AIN-C started, the widespread destruction from Hurricane Mitch in 1998 shifted how and where AIN-C was rolled out and expanded. Lastly, decentralization to municipalities took place during the rollout of AIN-C, though it is unclear what role this process played during the program’s implementation.

### Inputs

The right to health and food had been established in the constitution of Honduras, providing a legal expectation for supporting health and well-being [16]. Policy documents also cite other global-level commitments Honduras made to address hunger and its consequences, such as the World Food Summit and the Millennium Development Goals, as further basis for addressing malnutrition [16]. Unfortunately, the document review did not yield any information about financing, facilities, materials, equipment, or policies/guidelines for the implementation of AIN-C.

### System-level activities

#### Health system

Under *governance*, the MOH was committed to increasing coverage and quality of care, and empowering and incentivizing communities towards social control of nutrition programs [10, 16]. AIN-C was seen by policymakers and implementers at the time as a public demonstration of this commitment. Also, AIN-C was integrated with poverty reduction policies and the overall approach called for intersectoral coordination [16]. There was evidence of leadership from high-level government offices (e.g., Presidency, MOH), and high-level policies were put in place that supported AIN-C goals and integration with national child health programs [16]; however, there was more heated discussion about adding curative care components to the package of services delivered by *monitoras* ([12] and INT 08.15.14). Lastly, MOH decrees regarding AIN-C supported standardized implementation even when implemented by NGOs [11], but it was not clear who would monitor compliance.

In terms of *service delivery*, there were commitments and plans for extending the health system’s reach—again demonstrated by AIN-C. Also, CHWs appeared to be well integrated into the health system through their monthly visits to the health center for monitoring, supervision, and resupply.

Although there was considerable information on program costs, there was no information on program *financing* including funding sources and flows and timeliness. Likewise, there was no clear information on *health workforce* or any investment in MOH staff tasked with supervising *monitoras* and the program.

Regarding *information* activities, data at the individual level were collected in a simple, useful manner for *monitoras*, but it is not clear whether and how data were used by the MOH, either at the health facility level or higher up ([11]; INT 08.15.14). *Monitoras* were supposed to resupply with *medical products* and supplies at the health center during monthly supervision visits, but there was no information available on how well the supply chain was working.

#### Community

System-level activities from the community were not well represented in the documents, especially *governance/leadership* and *social belonging/cohesion*. There was limited information about active *resource mobilization* to address community issues that influence child growth, but it was anecdotal. Documents note that some community meetings facilitated by *monitora* teams to discuss growth monitoring data resulted in actions such as addressing contaminated water sources or trash sites, providing child care during busy times, improving indoor air pollution, and facilitating health center outreach [13].

### Program-level activities

#### Technical support

##### Health system

Most of the documentary sources were focused on program design and implementation. They highlighted several important features:

- Gradual rollout of AIN-C
- Formative research informed the program [10]:

- o Earlier experiences in Honduras with AIN
- o Evidence from other contexts (e.g., World Bank review of GMPs) [19]
- o In-country formative research (e.g., messages for counseling cards) [20]
- o Pilot testing

- There was an emphasis on strengthening initial training protocols, but investments in sustained training were unclear
- Supervision was operationalized to be standard throughout MOH facilities, but NGO implementers provided more supportive supervision

There was limited information about the ongoing monitoring and evaluation of the program. Few, if any, health centers used growth monitoring data to support *monitoras* or the community in their decision-making.

#### Community

There was no substantive information about technical support activities from the beneficiary communities.

#### Social support

##### Health system

AIN-C was designed to focus on the community’s capacity and responsibility to ensure that its children are fed and growing [10]. Various relationships necessary to support the program were facilitated by health sector actors, such as media advertising the program [10], and through integration of the *monitoras* and AIN-C into the health system, including defining roles and responsibilities [14]. However, there was no information about linkages between *monitoras* and other networks that could have supported them and AIN-C.

##### Community

In order to ensure community involvement and support, communities were advised that they needed to show their commitment by agreeing to join AIN-C and vest themselves in the program. This process was facilitated by engaging in conversations with communities and elders [15]. Further, they were tasked with selecting *monitoras*, and communities were given ownership over AIN-C materials (INT 07.30.14). It is not clear how well the quarterly community meetings were implemented, though, as noted above, there was anecdotal evidence of participatory decision-making leading to community actions [13].

#### Incentives

##### Health system

Program planners did not want to exceed the limits of the inherent volunteerism of *monitoras*, so health system incentives were planned for and operationalized [10]. Incentives from the health sector included identification cards, diplomas, carrying bags, letters of recognition/thanks from the Regional Health Office, yearly party/dinner, Children’s Day piñata parties, and preferential access to care at MOH facilities [12].

##### Community

There was some evidence that *monitoras* garnered stature and respect in the community due to their role, but this was not widely documented [14]. Anecdotal evidence suggests that *monitoras* became community leaders and advocates, especially after Hurricane Mitch when they helped mobilize communities to identify their needs and advocate to have them met (INT 07.30.14; INT 08.15.14; INT 09.10.14).

### Performance results

#### Outputs for CHW performance

An implementation review was conducted by BASICS after five full years of implementation [21]. Results of *monitora* performance indicated that:

- 17 % were making regular classifications errors
- 20 % were not counseling children with inadequate growth
- 60 % provided counseling with quality errors. Of these, 50 % were not using counseling cards correctly or at all

Challenges for counseling centered on correctly identifying specific problems linked to growth faltering and providing tailored advice. *Monitoras* were giving several, general recommendations to improve feeding practices instead of one or two targeted messages. Table 1 captures results from the implementation review regarding other issues affecting *monitora* performance.

**Table 1:** Implementation review findings on *monitora* performance

Performance category	Findings
Retention	• Average length of service for *monitoras* was 2.5 years. • 25 % of the original cohort was still working after 5 years. • *Monitoras* moved in and out of the program, which was facilitated by the team approach.
Motivation	• Active participation of beneficiary families was critical. • One third of *monitoras* noted the lack of family support.
Training	• Each community had at least one *monitora* who had participated in the original training. • However, 60 % of *monitoras* were learning by doing. • *Monitoras* in MOH communities received training on AIN-C and case management, while *monitoras* in NGO communities received additional training modules.
Supervision	• The content and quality of supervision varied. • Supervision was mainly focused on monthly health center meetings, but in NGO communities *monitoras* received additional supervision.
Supplies	• No stock-outs of basic materials were noted. • 90 % of scales used for weighing were accurate.
Data use	• 85 % of the child lists tracking children in the community were good. • Quality of progress bars tracking attendance and growth faltering depended on the quality of the child lists. • There was little use of bar charts by MOH for decision-making.
Community action	• Implementation was not uniform. • Community action depended on support from outside the community, with communities receiving support from health center promoters doing better. • Determined that about 20 % of causes for growth faltering that needed attention were issues outside the family.

Source [21]

#### Outcomes for client, community and health system change

A midterm evaluation of the AIN-C program was conducted in 2000 through a household survey that compared AIN-C communities with control communities served by the same health facilities [15]. A final evaluation was conducted in 2005 but was unable to use the same sample as earlier surveys due to issues of contamination of control communities and reduced implementation intensity [11]. Instead, the final evaluation took an individual-level approach to understand the impact of AIN-C by comparing children who participated in the program with those that did not, regardless of their community [11]. The evaluations explored topics around knowledge of and participation in a GMP, and knowledge, attitudes, and practices (KAP) at the household level. Table 2 shows results for the midterm evaluation comparing control and AIN-C communities, and results from the final evaluation comparing children in AIN-C and those not in a GMP program, as necessitated by the revised sample.

**Table 2:** AIN-C midterm and final evaluation results

	Baseline (1998)		Midterm (2000)		Final (2005)	
	Control (%)	AIN-C (%)	Control (%)	AIN-C (%)	No GMP (%)	AIN-C (%)
Child growth monitoring and promotion program awareness/participation						
Caregivers know about the GMP program in their community	7	27	15	96^a^	–	100
Caregivers participate in the GMP program in their community	21	30	23	92^a^	–	–
Enrollment in GMP program within first month of life	–	–	27	28	–	24
Caregiver has a growth card for child with at least two weight measurements	64	59	68	91^a^	–	93
Attend weighing session 3 or more time in past 3 months	38	30	44	70^a^	–	67
Caregiver received counseling for child with at least one instance of growth faltering on their growth card	–	–	57	81^a^	–	81
Caregiver recognition of counseling cards	–	–	31	64^a^	45	73
KAP around growth and feeding						
Exclusive breastfeeding of children under 6 months of age	15	21	13	39^a^	40	56^b^
Caregivers has their children 4 months of age or older take iron supplements	4	2	4	47^a^	30	66^b^
Caregiver aware that weight gain is sign of good growth	36	38	30	50^a^	33	51^b^
Caregiver aware that child being underweight is sign of poor growth	43	47	37	45^a^	41	48
KAP around illness						
Child is fully immunized by the age of 12 months	65	62	66	76^a^	71	77
Gave oral rehydration therapy to child with diarrhea	36	37	42	57^a^	38	62^b^
Gave child fluids and continued feeding during a bout of diarrhea	17	21	16	33^a^	70	82^b^
Child experienced in episode of diarrhea in past 2 weeks taken to *monitora* or health care provider	–	–	25	34	41	47
Child who experience episode of ARI in past 2 weeks taken to *monitora*, pneumonia volunteer or health care provider	–	–	44	36	–	–

Source [11, 15]

^a^Significant difference between AIN-C and control communities at midterm evaluation

^b^Significant difference between AIN-C and No GMP individuals at final evaluation

At midterm, caregivers in AIN-C communities showed improvements in their knowledge and practice despite poorer living conditions overall [15]. Participation in a GMP program was very high in communities targeted for AIN-C, with caregivers attending 70 % of weighing sessions regularly. In terms of KAP related to growth and feeding, AIN-C communities showed improvements over the control communities in many areas; however, very few caregivers in either group recognized inadequate weight gain as poor growth. Results for caregiving practices during illness were more mixed with limited gains in both groups. The final evaluation suggests stable rates of awareness and participation in the GMP program [11]. There were considerable improvements of KAP around growth and feeding and for care-seeking for diarrhea and ARI for both the AIN-C and No GMP groups. In the end, participation intensity was related to improvements in malnutrition: with every 1 % increase in participation, weight-for-age 0.005 z-score improved [11].

#### Impact

**Fig. 3:**
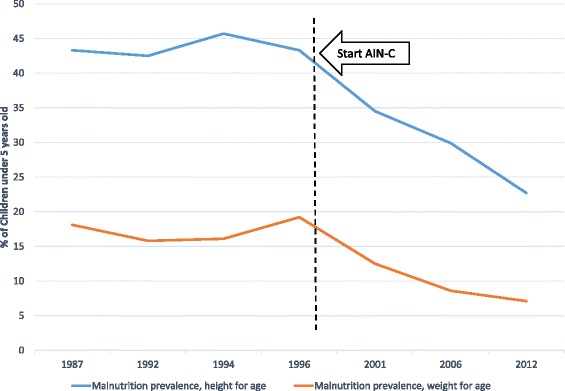
Malnutrition prevalence in Honduras, 1987–2012. Source [24]

**Fig. 4:**
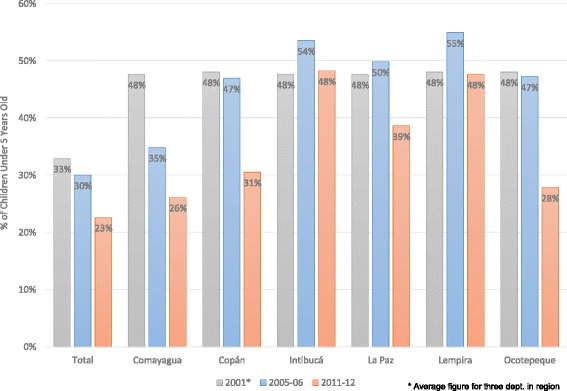
Height-for-age below two standard deviations in AIN-C departments, 2001–2011/12. Source [25–27]

National rates of malnutrition were already declining prior to the initiation of AIN-C, but the trend becomes more marked after its introduction (Fig. 3). However, decreasing national rates of malnutrition mask significant differences at the subnational level. While AIN-C departments have shown declines in malnutrition since 2001, their rates are still substantially higher than the national average (Fig. 4). These changes took place during a period of overall declines in population growth, fertility and child mortality, and improvements in life expectancy [22]. Management of malnutrition through GMPs is particularly challenging so it is difficult to ascribe success or failure directly to AIN-C but it appears that the program may have contributed to declines in malnutrition rates even though its direct effect cannot be measured.

## Discussion

This article describes a GMP delivered by CHWs in Honduras through the categories of the CHW performance logic model described by Naimoli et al. [9]. Results on the success of the program itself were mixed. An assessment of CHW performance indicated that many *monitoras* were providing effective counseling, but issues around classification errors, missed opportunities for counseling, and challenges in counseling quality were identified. In terms of health outcomes, evaluations of AIN-C found improvements in knowledge and caregiving practices and, most importantly, in malnutrition for children exposed to GMP with regular participation. However, due to the limitations of data available during this review, we were unable to draw direct linkages between components of the AIN-C program and positive health outcomes as intended. Below, we explore characteristics of AIN-C design that allowed the program to be contextually appropriate, reflect on the logic model itself, and raise considerations for future applications of the model.

In Table 3, we outline a number of characteristics of how AIN-C was designed to make the program better suited to the local community and health systems context, which can provide lessons to others designing similar programs. These have been broken down into three overarching categories: the content of the intervention, the delivery mechanism, and the mechanisms in place to support delivery.

**Table 3:** Critical characteristics of AIN-C emerging from program design

Characteristic of AIN-C	Design category^a^
Learning and formative research from earlier experiences informed program design	Content
Limit the education messaging per AIN-C visit	Content
Regularity of follow-up with program participants	Content
Targeting of worse-off communities	Delivery
CHWs working as a team sharing the workload	Delivery
Culture of volunteerism + operationalizing incentives	Delivery
Flexibility in implementation at the community level	Delivery
Information sharing up to the health system and down to the community	Delivery
Standardized plans for training, supervision and monitoring of CHWs	Support
Linkages between CHWs and health system: referrals, other services	Support
Community participation for site selection, CHW selection and community meetings	Support
Strong government and political commitment to the program	Support

^a^Design categories: content of the intervention, delivery mechanism, support structures

First, in terms of content, evidence was used by program planners to design AIN-C to avoid earlier pitfalls. The intervention was designed to focus on limited information per visit, with regular follow-up of participants. Second, there are several design characteristics related to the delivery of AIN-C worth noting. AIN-C was targeted at the communities who were the worst-off in order to realize the most gains and address equity. *Monitoras* were established as a team to allow CHWs to share the workload and reduce the likelihood of program collapse due to turnover. In order to avoid over-relying on the inherent volunteerism of community members, health sector incentives for *monitoras* were established. Further, flexibility in implementation and plans for information sharing allowed AIN-C delivery to be responsive to community needs.

Lastly, characteristics around supportive mechanisms covered both community and broader systems supports. On the community side, AIN-C communities had to agree to three main responsibilities: agreeing to become an AIN-C site, selecting *monitoras*, and holding community meetings. CHWs were linked with the health system through training, supervision, monitoring, and health referrals. Further, the strong government commitment to the overall efforts to address equity and improve health and well-being suggest high-level commitment to AIN-C’s goals.

### Reflections on the CHW performance logic model

First, we reflect on the ease of use of the CHW performance logic model and potential improvements to consider based on our experience and then address the potential uses suggested by the logic model’s authors.

Most monitoring and evaluation (M&E) efforts assessing CHW programs are not designed to assess CHW performance at the core of their activities or as a driver for the program’s theory of change. The CHW performance logic model is useful in helping reorient M&E approaches to focus more clearly on the intersection between CHW performance and program- and system-level activities. Consequently, our most important learning from using this logic model to evaluate AIN-C is that it is critical to start any evaluation with evidence on CHW performance as a way to provide a more focused approach to reviewing the program. While we were able to use the logic model to identify critical components of the program’s design, we were unable to identify factors contributing to programmatic success as we had originally intended.

Also, we identified several issues regarding the current composition of the logic model to consider for future iterations. First, the model as represented makes it appear that each component is equally weighted, when in reality this may be context-specific, which the authors acknowledge. Second, the technical support category is very broad and encompasses activities for multiple programmatic stages (e.g., design, implementation, evaluation), both for the health sector and community, resulting in a complex category that is unwieldy. Smaller categories would help clarify the potential roles of different actors to identify and address gaps, and strengthen the program. Similarly, the inputs and context categories are broad and include many complex components, such as policies, funding, organizations, which have likely implications for program success. In fact, a recent review proposed an approach stressing the critical pathways through which contextual factors influence CHW performance [23]. Third, key aspects of the AIN-C program beyond CHW performance were hard to locate in the logic model, such as government and political commitment, cost of the program, flexibility in implementation as a design feature, and commitments to equity, which highlight the importance of factors external to CHWs in supporting both their performance and ultimate outcomes.

#### Potential uses of the CHW performance logic model

The logic model authors suggested four potential uses for the CHW performance logic model, each with their own considerations, which we address in turn. For planning, the logic model may be too comprehensive with many components to focus on, which could overwhelm policymakers. It would be helpful to identify which are the key categories to focus on at the outset of planning a program, or provide guidance for a facilitated planning process with the logic model as its basis. For practical purposes, the logic model could be used to (i) assess the current programmatic landscape and the potential contributions of a new intervention delivered by CHWs, (ii) explore how program- and system-level activities may support or hinder the CHW program and meeting its goals, and (iii) for careful reflection on community contributions.

For consensus building, the logic model can be used through guided discussions aiming for a coordinated approach; the caveat above about the number and complexity of categories would also be a concern here. For program implementation, the model could be used to inform discussions around prioritizing investments and problem solving. However, it could be difficult to tease out specific areas to address without targeted and regular monitoring data. As for evaluation, the model identifies all the potential categories necessary to develop a comprehensive evaluation design. In fact, we believe that Honduras would benefit greatly from an on-the-ground evaluation of the AIN-C program using the CHW performance logic model to identify and address program challenges.

Lastly, we stress an important consideration for these strategies: communities. A clear role for communities would need to be identified a priori when using the logic model for these strategies to ensure that programs are planned, built, implemented, and evaluated in a representative, transparent, and responsive manner.

### Limitations

There are several limitations to this review. First, there was limited documented information about AIN-C available in the public domain. Most of the available evidence focused on the period of BASICS and USAID support, and minimal publicly available information on the current status of AIN-C. We attempted to address this limitation by conducting additional literature review searches through databases of published research as well as general web searches, but few additional documents were identified. Second, for interviewees, there had been a lag of about 10 years between their participation in AIN-C and their interview for this project, which may have limited their recall of details on the program. Lastly, in terms of the application of the logic model itself, the core documents about AIN-C contain little discussion about the community component of the program making it difficult to ascertain its contributions despite the intentions in the design and original rollout. We are unsure whether the lack of a documented community role in AIN-C represents poor community involvement or uneven documentation.

## Conclusions

This retrospective desk review explored a GMP in Honduras delivered by CHWs through the lens of a CHW performance logic model. It identifies lessons to be learned from the program’s design as well as from the potential of the logic model itself, which provides a comprehensive basis for understanding, planning, and evaluating CHW programs into the future.

## Additional file

Additional file 1: Supplementary file. (PDF 98 kb)
