# Supplementary material for: A retrospective review of the Honduras AIN-C program guided by a community health worker performance logic model
Source: Hum Resour Health. 2016 May 6;14:19. doi: 10.1186/s12960-016-0115-x (PMC4858906; doi:10.1186/s12960-016-0115-x)
Supplement: Additional file 1: — Supplementary file. (PDF 98 kb) [file 12960_2016_115_MOESM1_ESM.pdf]

A retrospective review of the Honduras AIN-C program guided by a community health worker performance logic model – Supplementary File

Documents Reviewed

BASICS II. (2003, Dec 2003). *June 2003 Process Review Data from Community AIN in Honduras.* Paper presented at the TEG Meeting.

Consorcio Gesaworld Group-Econometría S.A. (2013). Informe Final de la Consultoría de Evaluación Externa de Impacto de la Estrategia Atención Integral a la Niñez en la Comunidad (AIN-C). Tegucigalpa: Secretaría de Salud de Honduras.

Corrales, G., Pavón, S., Enamorado, R., Stupp, P., Jefferds, M., Grummer-Strawn, L., . . . Monteith, R. (2002). Honduras Encuesta Nacional de Epidemiología y Salud Familiar / Encuesta Nacional de Salud Masculina 2001 - Informe Final. Honduras: Secretaría de Salud.

Fiedler, J. (2003). A Cost Analysis of the Honduras Community-based Integrated Child Care Program (Atención Integral a la Niñez-Comunitaria, AIN-C) *Health, Nutrition and Population Discussion Paper*. Washington, DC: World Bank.

Fiedler, J. L., Villalobos, C. A., & De Mattos, A. C. (2008). An activity-based cost analysis of the Honduras community-based, integrated child care (AIN-C) programme. *Health Policy Plan, 23*(6), 408-427. doi: 10.1093/heapol/czn018

González Carías, S. Interculturalidad de la Salud en Poblaciones Indígenas Lenca y Maya-Chortí de Honduras: Prácticas alimentarias y terapéuticas tradicionales en mujeres y niños y niñas menores de cinco años en poblaciones Lenca y Maya-Chortí de Honduras. Honduras: Secretaría de Salud de Honduras.

Griffiths, M. Atención a la Niñez en la Comunidad (AIN-C) Monitora Strategy: The Manoff Group.

Griffiths, M. Promoting the Growth of Children and the Education of Families and Communities *Just the BASICS*. Washington, DC: USAID - BASICS II.

Griffiths, M. (2004). *Implementación: El Vínculo Entra Teoría y los Resultados?* Presentation. USAID-BASICS II.

Griffiths, M. (2010a, 4-5 Nov 2010). *El modelo: Promoción de Crecimiento con Base Comunitario (PCBC).* Paper presented at the Diálogo Regional de la Red de Protección Social.

Griffiths, M. (2010b, 26 April 2010). *Using growth to integrate and organize community nutrition programming: Selected topics.* Paper presented at the WB Nutrition Retreat.

Griffiths, M., & Del Rosso, J. (2007). Growth Monitoring and the Promotion of Health Young Child Growth: Evidence of Effectiveness and Potential to Prevent Malnutrition: The Manoff Group.

Griffiths, M., Dickin, K., & Favin, M. (1996). Promoting the Growth of Children: What Works - Rationale and Guidance for Programs *World Bank Nutrition Toolkit* (Vol. Tool 4). Washington, DC: Human Development Dept. - The World Bank.

Griffiths, M., & McGuire, J. S. (2005). A New Dimension for Health Reform - The Integrated Community Child Health Program in Honduras. In G. M. LaForgia (Ed.), *Health Systems Innovations in Central America: Lessons and Impact of New Approaches*. Washington. DC: The World Bank.

Honduras Integrated Child Health Care: The AIN Community Experience. USAID BASICS.

Honduras Workplan FY2004. (2004). from BASICS

Marini, A., Bassett, L., Bortman, M., Flores, R., Griffiths, M., & Salazar, M. (2009). Promocion del crecimiento para prevenir la desnutrición crónica: Estrategias con Base Comunitaria en Centro América. Washington, DC: World Bank,.

Martínez, R., & Fernández, A. (2007). El costo del hambre: impacto social y económico de la desnutrición infantil en Centroamérica y República Dominicana. Santiago de Chile,: Comisión

Económica para América Latina y el Caribe (CEPAL), Programa Mundial de Alimentos - Naciones Unidas.
Pearson, A. M., Griffiths, M., McCarthy, D., & Contreras, A. (2002). BASICS Honduras Country Program: Final Report on Technical Activities Carried Out with Reconstruction Funds: The Manoff Group.
Proyecto Nutrición y Protección Social: Evaluación Social. (2005): Secretaría del Despacho Presidencial.
Schaetzel, T. Enabling Community Workers and Mobilizing Communities - Communications Strategies of the AIN-C Health and Nutrition Programme: USAID's Infant and Young Children Nutrition Project,.
Schaetzel, T., Griffiths, M., Miller Del Rosso, J., & Plowman, B. (2008). Evaluation of the AIN-C Program in Honduras. Arlington, VA: Basic Support for Institutionalizing Child Survival Project (BASICS II) for USAID.
Secretaría de Estado en el Despacho de Salud. (2005). *Plan Nacional de Salud 2021*. Tegucigalpa: República de Honduras.
Secretaría de Salud. (2005a). *Política Nacional de Nutrición*. Honduras: República de Honduras,.
Secretaría de Salud. (2005b). *Política Nacional de Salud Materno Infantil*. Honduras: República de Honduras,.
Secretaría de Salud. (2010). *Plan Nacional de Salud 2010-2014*. Honduras: Secretaría de Salud.
Secretaría de Salud. (2013). *"Por una Honduras Saludable": Modelo Nacional de Salud*. Tegucigalpa: Secretaría de Salud.
Secretaría de Salud [Honduras], Instituto Nacional de Estadística, & ICF International. (2013). Encuesta Nacional de Salud y Demografía 2011-2012. Tegucigalpa, Honduras: SS, INE e ICF International.
Secretaría de Salud [Honduras], Instituto Nacional de Estadística, & Macro International. (2006). Encuesta Nacional de Salud y Demografía 2005-2006. Tegucigalpa, Honduras: SS, INE y Macro International.
Van Roekel, K., Plowman, B., Griffiths, M., Vivas de Alvarado, V., Matute, J., & Calderón, M. (2002). BASICS II Midterm Evaluation of the AIN Program in Honduras, 2000. Arlington, VA: Basic Support for Institutionalizing Child Survival Project (BASICS II) for USAID.
